# Ground-Target Recognition Method Based on Transfer Learning

**DOI:** 10.3390/s25020576

**Published:** 2025-01-20

**Authors:** Qiuzhan Zhou, Jikang Hu, Huinan Wu, Cong Wang, Pingping Liu, Xinyi Yao

**Affiliations:** 1 College of Communication Engineering, Jilin University, Changchun 130012, China; zhouqz@jlu.edu.cn (Q.Z.); hujk18@mails.jlu.edu.cn (J.H.); wangcong2020@jlu.edu.cn (C.W.); xinyiy19@mails.jlu.edu.cn (X.Y.); 2 College of Computer Science and Technology, Jilin University, Changchun 130012, China; liupp@jlu.edu.cn

**Keywords:** target recognition, convolutional neural network, domain adaptation, transfer learning, vibration sensors

## Abstract

A moving ground-target recognition system can monitor suspicious activities of pedestrians and vehicles in key areas. Currently, most target recognition systems are based on devices such as fiber optics, radar, and vibration sensors. A system based on vibration sensors has the advantages of small size, low power consumption, strong concealment, easy installation, and low power consumption. However, existing recognition algorithms generally suffer from problems such as the inability to recognize long-distance moving targets and adapt to new environments, as well as low recognition accuracy. Here, we demonstrate that applying transfer learning to recognition algorithms can adapt to new environments and improve accuracy. We proposed a new moving ground-target recognition algorithm based on CNN and domain adaptation. We used convolutional neural networks (CNNS) to extract depth features from target vibration signals to identify target types. We used transfer learning to make the algorithm more adaptable to environmental changes. Our results show that the proposed moving ground-target recognition algorithm can identify target types, improve accuracy, and adapt to a new environment with good performance. We anticipate that our algorithm will be the starting point for more complex recognition algorithms. For example, target recognition algorithms based on multi-modal fusion and transfer learning can better meet actual needs.

## 1. Introduction

The security of important areas (such as ancient tombs, military bases, infrastructure, and residential areas) is a necessary means to ensure social stability and the normal life of residents. The placement of moving ground-target identification systems based on vibration sensors at national borders and key areas is important for maintaining regional security and stability. In the intelligent security system, it is very important to analyze the vibration signal and study the recognition algorithm of the moving ground target. The ground-target movement process will continue to generate vibration signals propagated in the shallow ground surface. The analysis of vibration signals is an effective means of identifying the type of target. Devices to realize vibration sensing mainly include optical fibers [1,2], detection cables [3], and vibration sensors [4,5]. Among them, the moving ground-target recognition system based on vibration sensors has the advantages of small size, low power consumption, and strong concealment, which are of great research significance in various fields. Current target recognition algorithms are often unable to adapt to changes in new environments. Different regions have different environmental noise. Vehicles and humans produce different signals. This leads to large differences in the recognition accuracy of the same model in different environments. Therefore, we need to study a target algorithm based on the existing vibration-sensing equipment. This algorithm can accurately identify the target type and adapt to environmental changes. Simultaneously, we used vibration sensors to collect the vibration signals. A vibration sensor-based recognition system can reduce power consumption.

Y.W et al. [6] proposed a new method for accurate real-time detection and identification of multiple vibration events in perimeter safety air-space integration. This method utilizes a distributed optical fiber sensor and the YOLO method. Y.L et al. [7] proposed a new approach for radar ground target recognition based on semantic guidance and hierarchical classification. The subtask is matched with the optimal feature and local classifier to realize target recognition. However, most of these methods are based on radar or fiber-optic signals. This results in increased energy consumption in the system. The target recognition algorithm based on the decision mechanism relies mainly on the different characteristics of different types of vibration signals for design. Smith J. F. [8] designed a fuzzy-logic boundary object classification algorithm. This method extracts features such as the rhythm, orientation, power, and kurtosis of the target signal from the vibration signal and constructs a recognition model using a decision tree. Sindhu N. et al. [9] combined wavelet transform and symbolic dynamic filtering to realize vehicle classification. In the case of imperfect datasets, recognition algorithms based on decision mechanisms often cannot be generalized. Simple classifier-based target recognition algorithm inputs low dimensional feature vectors of vibration signals. Du K. et al. [10] used a multi-scale generalized fractal dimension matrix to characterize the vibration signals generated by the motion of wheeled and tracked vehicles. Train a vehicle recognition model using a support vector machine (SVM). Kalra M. et al. [11] used empirical wavelet transform to construct a vehicle classification model to recognize buses and tractors. The fitting ability of simple classifiers is very limited; therefore, the use of neural networks to fit high-dimensional features for target recognition has received considerable attention. Jin G. et al. [12] constructed a neural network based on signal time–frequency characteristics to achieve ground vehicle recognition. The network takes the log-scaled frequency cepstral coefficient matrix of vehicle vibration signals as input and outputs the recognition result as a wheeled or tracked vehicle through a CNN. Bin K. et al. [13] used CNN to construct an edge-oriented intelligent target recognition method to distinguish between pedestrian vibration signals and vehicle vibration signals. However, these studies have not adapted well to the environment. In a new environment, recognition accuracy is reduced.

Most moving ground-target recognition algorithms have some drawbacks: (1) they cannot adapt to the new environments, and (2) most target recognition models based on neural networks have complex structures and are difficult to train to converge. Therefore, to solve the above problems, we propose a new method of moving ground-target recognition, named domain adaptive target recognition (DATR). The algorithm (1) introduces transfer learning [14] to adapt to environmental changes, and (2) reduces the model complexity to reduce the training and running times. Compared with the present target recognition algorithm, this algorithm can improve recognition accuracy. In the new environment, the recognition accuracy of this algorithm can still be maintained at a relatively high level.

## 2. Methodology

### 2.1. Ground-Target Recognition Method

**Figure 1:**
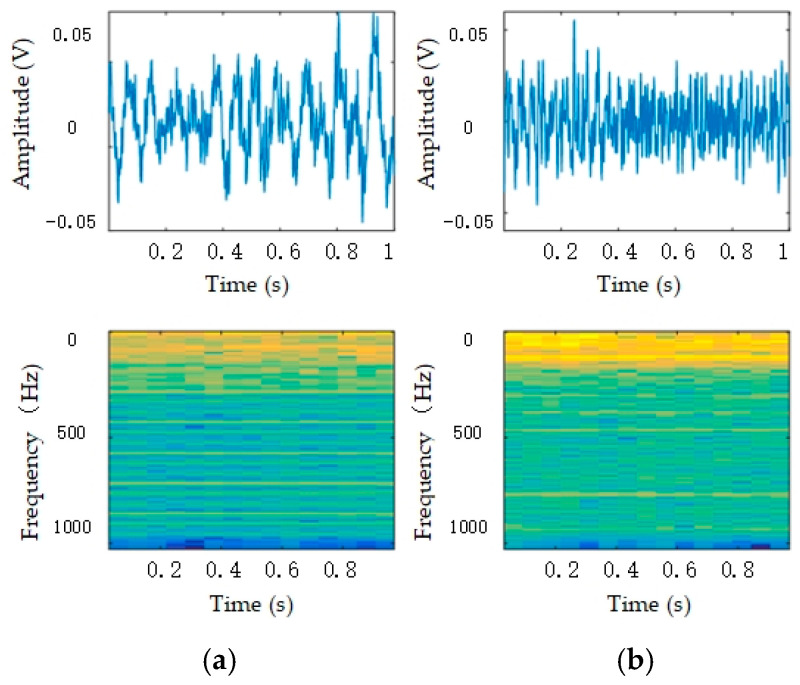
Time-domain and time–frequency domain of vehicle signal: (**a**) tracked vehicle; (**b**) wheeled vehicle.

The time-domain and time–frequency domain of the vehicle signal are shown in Figure 1. It can be observed that the waveform representation of different types of vehicle signals in the time-domain signal is continuous. The energy in the frequency domain of the tracked vehicle was concentrated in a higher frequency band than that of the wheeled vehicle. In addition, the energy variation of the vehicle vibration signals at different frequencies exhibited different trends over time.

**Figure 2:**
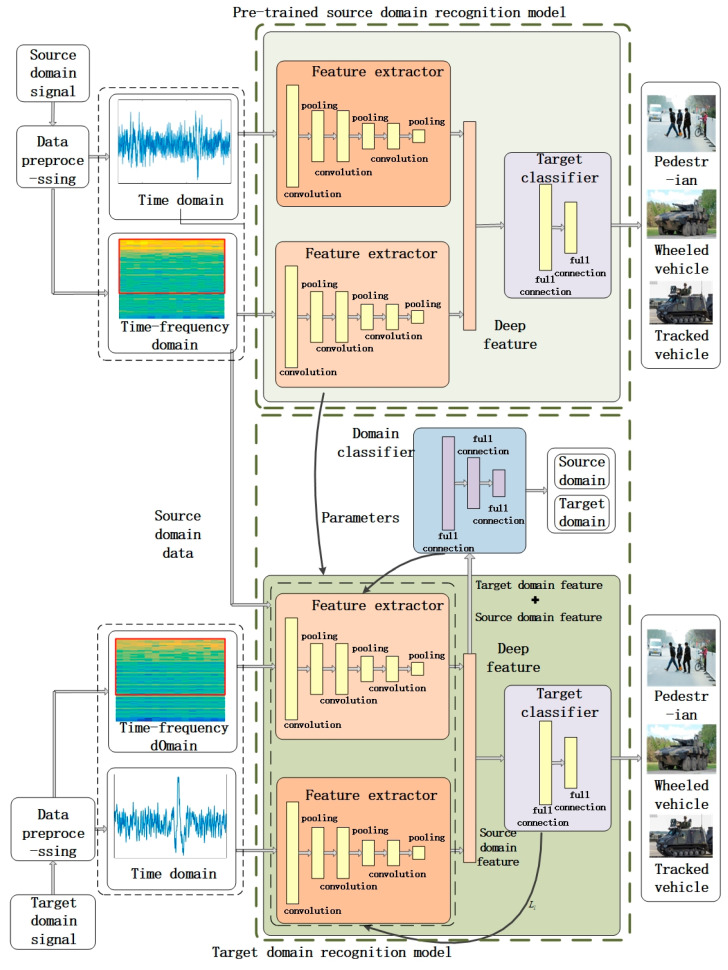
Overall architecture.

We propose a DATR algorithm based on transfer learning and neural networks for recognizing moving ground target, such as pedestrians, wheeled vehicles, or tracked vehicles. The algorithm takes the time-domain waveform and the time–frequency map of the signal as input. The recognition model was constructed using CNN to extract deep features. The extracted deep features were identified in the target classification. The overall architecture is shown in Figure 2.

The source-domain target recognition model consists of two feature extractors and a target classifier. They are used to learn the features of the source-domain target vibration signals, which helps construct the base recognition model and provide initialization parameters for the target-domain recognition model. Feature extractor 1 is used to extract features from the time–domain waveform of the signal. Feature extractor 2 is used to extract features from the time–frequency representation of the signal. The deep features extracted from the signal by the feature extractor are fed into the target classifier training to construct the source-domain recognition model. The source-domain model is migrated to the target domain model as a priori knowledge to assist in the training. The parameters of the source-domain recognition model correspondingly migrated to the target domain model to complete the network weight initialization.

The target–domain recognition model consists of two target domain feature extractors, a target classifier, and a domain classifier. The inputs to the feature extractor are labeled as vibration signals in the source domain and unlabeled vibration signals in the target domain. The deep feature matrices of the signals extracted by the target domain feature extractors are fed into the target classifier and domain classifier to perform model training. The deep features of the vibration signals in the source domain and the corresponding category labels are input to the target classifier. The feature matrix of the vibration signals in the source domain, the feature matrix of the vibration signals in the target domain, and domain labels corresponding to the feature matrices are input into the domain classifier.

In the gradient update of the target domain model training, the loss of the target classifier and the loss of the domain classifier are passed back to the feature extractor for network parameter fitting. The feature extractor aims to extract the common features of the source and target-domain signals to achieve target identification. Thus, the domain classifier cannot distinguish between source and target-domain features. Simultaneously, the target classifier outputs the signal class correctly. The training objective of the feature extractor is to maximize the loss of the domain classifier while minimizing the loss of the target classifier. The domain classifier aims to differentiate the features extracted by the feature extractor from the source and target-domain signals. The training objective is to minimize the domain classification loss. During the model training process, a lower learning rate is used to iteratively tune the parameters of each layer of the recognition model. Stop until the number of iterations reaches the set epoch value or the loss function satisfies the early stop condition.

The adversarial training method between the feature extractor and the domain classifier reduces the parameters of the feature extractor. It is updated to reduce the difference between the distribution of features extracted from the source domain and features extracted from the target domain. The trained feature extractor can extract the common features of the vibration signals in the source and target domains. Thus, the recognition model has a higher recognition accuracy for the target-domain data in the new environment.

### 2.2. Algorithm Model Structure

The algorithm proposed in this paper consists of two feature extractors, a target classifier, and a domain classifier. The structure of each part is as follows.

**Figure 3:**
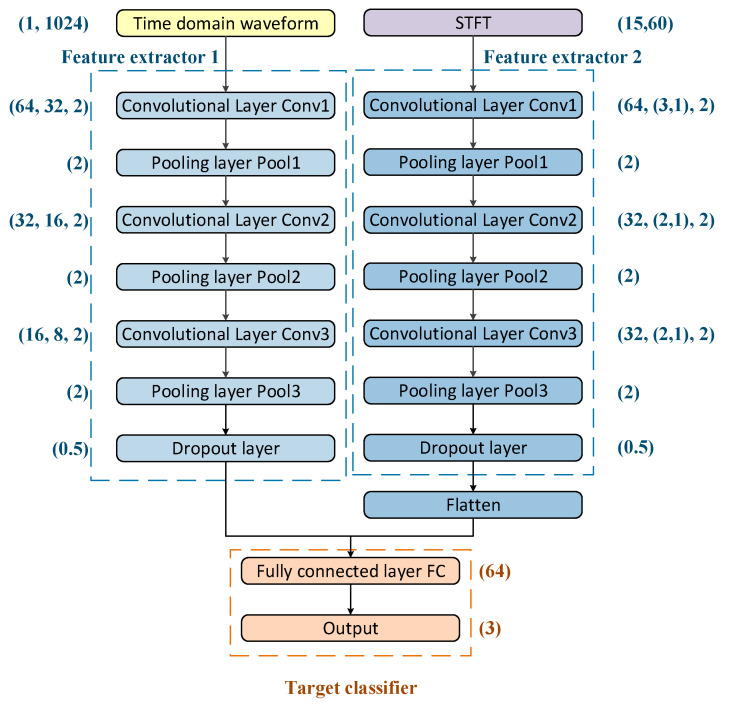
The structure of the recognition model.

The structure of the recognition model feature extractor and target classifier is shown in Figure 3. The input identification of feature extractor 1 is (number of channels, data length). The input identification of feature extractor 2 is expressed as (data length, data width). The convolutional layer is identified as (number of channels, convolutional kernel size, step size). The pooling layer is identified as (pooling length) and the fully connected layer is identified as (number of nodes). The recognition model output identification is (number of target categories).

**Figure 4:**
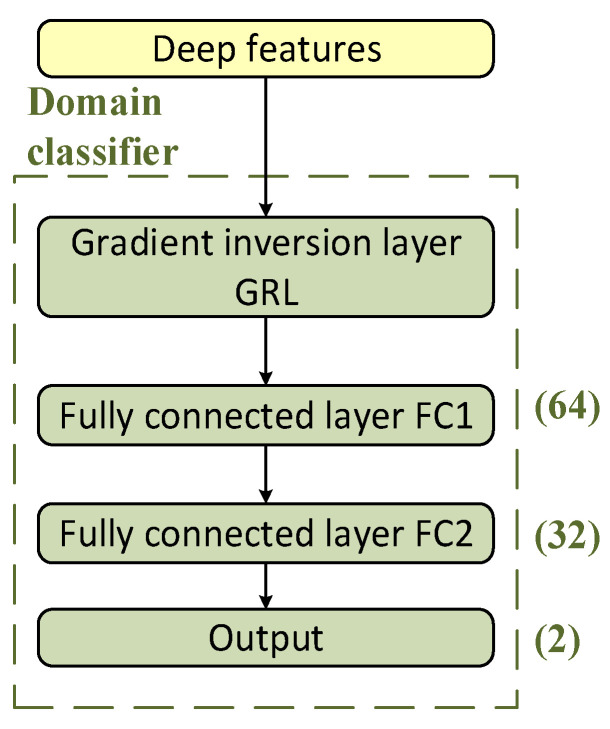
The structure of the domain classifier.

The structure of the domain classifier is shown in Figure 4. The fully connected layer is identified as (number of nodes) and the output is identified as (number of domain categories). The domain classifier is constructed by the fully connected layer to make a combination judgment on the deep features of the original signal and construct the adversarial training between it and the feature extractor.

The input to the domain classifier is the deep feature matrix of the signal output by the feature extractor. The loss gradient of the network training time-domain classification and the gradient update direction of the target recognition loss are opposite. Therefore, it is necessary to add a gradient reversal layer (GRL) before the domain classifier fully connects layer FC1. This can realize an inverse gradient in the backpropagation process from the domain classifier to the feature extractor. It can also realize the adversarial training of the feature extractor and the domain classifier. The GRL formula is expressed as follows:

(1) $$\begin{array}{c} R_{\lambda }\left(x\right)=x\\ \frac{dR_{\lambda }\left(x\right)}{dx}=-\lambda I \end{array}$$

where $\lambda =\frac{2}{1+e^{-\gamma \cdot p}}-1$, γ is a hyperparameter; p is the number of current training steps divided by the total number of steps; I is the unit matrix.

### 2.3. Model Loss Function

The loss of source-domain model training is evaluated using the cross-entropy loss function for multiple classifications, as shown in the following equation:

(2) $$L_{l_{S}}=-\sum_{i=1}^{C_{s}}y_{i}\log(p_{i})$$

where $C_{s}$ is the number of categories of the source-domain vibration signal; $y_{i}$ is the sign function; If the true category of the sample is i then $y_{i}=1$, otherwise $y_{i}=0$. $p_{i}$ is the predicted probability that the sample belongs to the category i.

The loss of the target classifier is represented by the cross-entropy loss function for multi-categorization as shown in the following equation:

(3) $$L_{l_{T}}=-\sum_{i=1}^{C_{t}}y_{i}\log(p_{i})$$

where $C_{t}$ is the number of target-domain vibration signal categories.

In order to prevent the imbalance between the number of training samples in the source and target domains of training leads to excessive model skewing, the focal loss was used to construct the antagonistic loss between the source-domain and target–domain data in domain classifier training. This will increase the proportion of target-domain samples in training and suppress the impact of sample imbalance on model training. The domain classifier loss constructed by focal loss is shown in the following equation:

(4) $$\begin{array}{c} L_{d}=-\alpha_{t}{\left(1-p_{t}\right)}^{\gamma_{d}}\log\left(p_{t}\right)\\ p_{t}=\left\{\begin{array}{cc} p & ,\;y=1\\ 1-p & ,\;y=0 \end{array}\right.\\ \alpha_{t}=\left\{\begin{array}{cc} \beta & ,\;y=1\\ 1-\beta & ,\;y=0 \end{array}\right. \end{array}$$

where p is the predictive probability of the model; ${\left(1-p_{t}\right)}^{\gamma_{d}}$ represents the modulation factor; $\gamma_{d}$ is an adjustable coefficient that reduces the loss of easy-to-learn samples (source domain samples) and increases the weight of target domain samples in model learning.

The MMD (Maximum Mean Discrepancy) distance measures the distance between the distributions of two feature vectors. For the source-domain–time-domain waveform feature set $F_{st}$ and the target-domain–time-domain waveform feature set $F_{tt}$, the MMD distance is defined as shown in the following equation.

(5) $$MMD_{t}(F_{st},F_{tt})={\left\|\frac{1}{n}\sum_{i=1}^{n}\phi (f_{i_{st}})-\frac{1}{m}\sum_{i=1}^{m}\phi (f_{i_{tt}})\right\|}_{H}$$

where $f_{i_{st}}$ is the signal time-domain features within the set $F_{st}$; $f_{i_{tt}}$ is the signal time-domain features within the set $F_{tt}$; n is the number of source-domain features; m is the number of target-domain features; ϕ(⋅) is a nonlinear mapping from the original feature space to the regenerated Hilbert space; H is the regenerated Hilbert space.

For the source-domain–time-domain waveform feature set $F_{ss}$ and the target domain–time-domain waveform feature set $F_{ts}$, the MMD distance is defined as shown in the following equation:

(6) $$MMD_{s}(F_{ss},F_{ts})={\left\|\frac{1}{n}\sum_{i=1}^{n}\phi (f_{i_{ss}})-\frac{1}{m}\sum_{i=1}^{m}\phi (f_{i_{ts}})\right\|}_{H}$$

In summary, the overall loss function of the recognition network in this paper is shown in the following equation:

(7) $$L_{t}=L_{l_{T}}+L_{d}+\alpha \left(MMD_{t}(F_{st},F_{tt})+MMD_{s}(F_{ss},F_{ts})\right)$$

where α is the proportionality parameter for that portion of the loss.

## 3. Experimental Setup and Result Analysis

### 3.1. Experimental Setup

The target vibration signals in the dataset were divided into three groups, dataset A, dataset B, and dataset C, to validate the performance test of the target recognition algorithm. The experiment was realized on a computer with 64-bit Win7 system, i5-7500 (CPU 3.4 GHz) processor, and 8.00G RAM.

The data set is the vibration data that we collect from different regions using vibration sensors. Environmental noise are data collected without any moving ground targets. Pedestrian signals are data on the movement of pedestrians in the monitored area. Tracked vehicle data and wheeled vehicle signals are the data of two different types of vehicles driving in the monitored area. Due to the limitations of the data acquisition environments and test vehicle types, some environments did not capture the data for all target types. Therefore, the ambient noise at the signal acquisition locations was compared, and vibration signals from locations with similar ambient noise characteristics were selected as a set of data sets. The sample data settings for the three datasets are shown in Table 1, and the length of each sample is 1 s.

The input to the feature extractor is a time-domain waveform of length 1024. The sampling rate of the target vibration signal in the sample database is 2048 Hz. Although the dense waveform points can better represent the variation of the time-domain waveform, it leads to an increase in model computation. In order to reduce the computation of the feature extraction network, the original vibration model is downsampled to reduce the length of the input as much as possible while preserving the time-domain waveform characteristics of the signal. After experimental testing, the sampling rate of the data was reduced to 1024 Hz.

The input to the Short-Time Fourier Transform (STFT) of the signal is a vibration signal with a length of 1024, with a single window of 0.125 s and an overlapping window of 0.0625 s. The size of the STFT time-frequency representation obtained is 15 × 256. In order to reduce the amount of network computation, the effective frequency of the target vibration signal in the time–frequency representation is selected range to construct the feature map. Then, the feature map is converted into a grayscale image and input into the CNN. According to the energy band distribution of the target vibration signal, the low-frequency part of the time–frequency representation is selected to characterize the signal, and the input of the feature map is set to 15 × 60.

In the recognition model, the ReLU function is selected as the activation function of the convolutional layer. The softmax function is selected as the output function of the fully connected layer. In addition, the dropout layer is used to improve the generalization ability of the model and its ratio is set to 0.5.

In the loss function, $C_{s}=C_{t}=3$, β=0.25, $\gamma_{d}=2$, α=0.2.

The target recognition algorithm DATR proposed in this paper is experimentally compared with VibCNN [15] and PRNN [16]. The parameters setup is consistent with the related papers. Among them, VibCNN takes the 1D time-domain waveform of the vibration signal as input, and uses CNN to construct the recognition model. The inputs of PRNN are the time-domain waveform and spectrum of the signal, and the recognition model is constructed by using the LSTM network. The settings for model training in this paper are shown in Table 2, and an early stopping mechanism is adopted during network training to avoid network over-fitting.

### 3.2. Experiments and Comparative Analysis of Base Model Performance

#### 3.2.1. Recognition Performance Comparisons

This subsection compares the recognition performance of this algorithm with other algorithms. The performance of the recognition algorithm is evaluated using Macro-Accuracy and Macro-F1-score. This subsection describes the experiments that show the accuracy of the proposed method for target recognition.

Dataset A, shown in Table 1, is used to train the recognition model and compare the recognition performance of the DATR algorithm proposed in this paper with the VibCNN and the PRNN. Dataset A includes three target categories: pedestrian vibration signal, wheeled vehicle vibration signal and tracked vehicle signal. The proportions of training samples, validation samples and test samples are set to 60%, 20%, and 20%*,* respectively. Data were randomly selected from the dataset proportionally for 25 experiments, and the average accuracy and confidence interval of the experiments were calculated. The performance of the three methods is shown in Table 3.

From Table 3, it can be seen that the average Accuracy of the DATR algorithm on the test set is improved by about 1.2–2.8%. The average F1-score is improved by about 1.1–2.9% compared with the comparison method, and it has the best recognition performance on both the validation set and the test set. Comparing the average accuracies and confidence intervals of the different algorithms for multiple repetitive experiments, it can be seen that the range of variation of the accuracy of the DATR algorithm is smaller than that of the VibCNN algorithm. It shows that its algorithm stability is better than the VibCNN algorithm, and the average accuracy of the VibCNN algorithm is slightly lower than the DATR algorithm. Due to the complex structure of the recognition network of the VibCNN algorithm, more network layers may lead to gradient instability in model training and cause the accuracy of the recognition model to decrease. Compared with the PRNN algorithm, the DATR algorithm has a higher recognition accuracy and F1-score, and can more accurately distinguish between human targets, wheeled vehicle targets, and tracked vehicle targets. From the model testing results, the CNN-based network has better fitting ability than the LSTM-based network.

In summary, the DATR algorithm has higher recognition accuracy and better model training stability than the VibCNN algorithm and the PRNN algorithm.

#### 3.2.2. Different Target Recognition Performance

This subsection describes the experiments that show the accuracy of the proposed method for identifying different types of targets.

**Figure 5:**
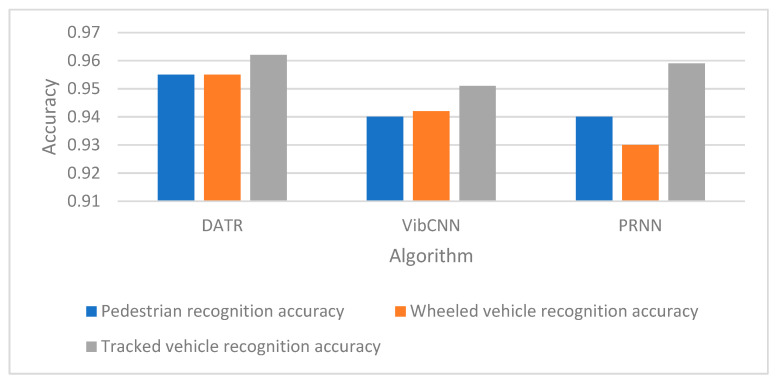
Identification accuracy.

The following tests the recognition performance of the DATR algorithm and comparison methods for different types of targets. The data test set is used to verify the recognition performance of the three methods for pedestrians, wheeled vehicles and tracked vehicles. The identification accuracy of the three methods are shown in Figure 5. The recognition accuracies of the DATR algorithm for the three types of targets of pedestrians, wheeled vehicles and tracked vehicles are 95.5%, 95.5%, and 96.2%, respectively. The DATR algorithm has high recognition accuracies on the task of recognizing all types of targets, and there is no problem that the recognition methods do not have an uneven recognition accuracy for multi-targets.

The experimental results show that the feature extractor of DATR algorithm can extract better target features of differentiation, recognize different types of targets, and have high recognition accuracy. The algorithm has high recognition accuracy on the task of recognizing all types of targets, and there is no problem with uneven recognition accuracy of multiple targets by recognition methods.

#### 3.2.3. Computing Efficiency

This subsection describes the experiments that show the computational efficiency of the proposed method.

In order to evaluate the computing efficiency of the classification algorithms, the training time and running time of the three recognition algorithms are compared, and the results are shown in Table 4.

From the table, we can see that the training time of DATR is 11 min 34 s. With the same number of epochs, DATR takes about one-seventeenth of the training time of the VibCNN method (the time shown in the table is the time required for VibCNN to run 200 epochs). The single judgment operation time of DATR is 0.8 ms on average, which is 3.6 ms faster than the single judgment operation time of the VibCNN algorithm. From the above experimental results, it can be seen that the DATR algorithm not only has higher recognition accuracy but also has faster model training time and computation time. In practical applications, the DATR algorithm has higher judgment efficiency, which can provide real-time recognition results for personnel and reduce system power consumption.

### 3.3. Experiments and Comparative Analysis of Recognition Algorithm Performance

#### 3.3.1. Identify Manifestations Across Domains

This subsection shows the experiments that show the performance of unmigrated DATR and other algorithms in the new environment.

**Figure 6:**
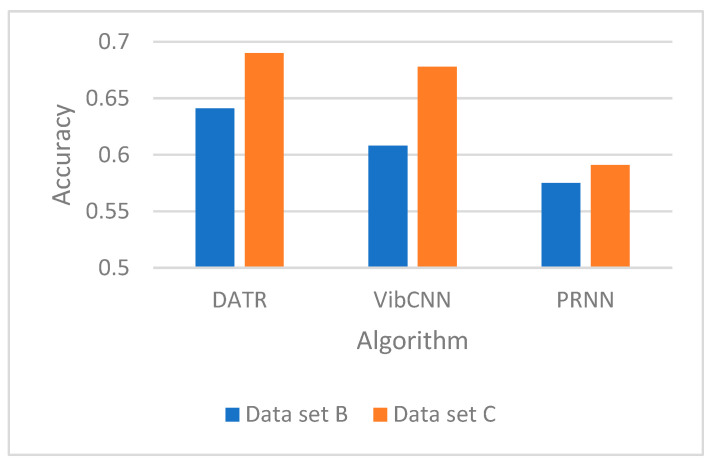
Model accuracy.

**Figure 7:**
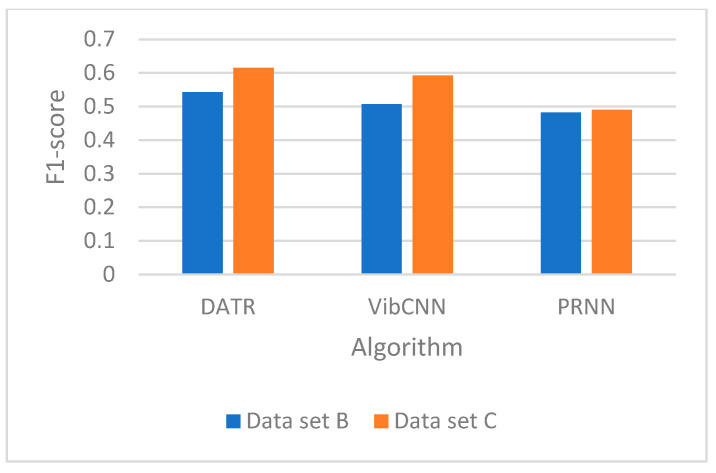
Model F1-score.

We test the performance of the source-domain recognition model in the new environment. The source-domain recognition model is trained using dataset A, and its recognition accuracy on target=domain datasets B and C is tested and the experimental results are shown in Figure 6 and Figure 7.

As can be seen from the figure, the accuracy of DATR as well as VibCNN and PRNN is 64%, 60%, and 57% for dataset B and 69%, 68%, and 59% for dataset C, respectively. The decrease in accuracy of this recognition model in a new environment (new dataset) is due to the change in target features. The original recognition model is not able to achieve high performance on the new dataset due to the changes in the characteristics of the target vibration signals caused by changes in the environment and changes in the vehicle type.

#### 3.3.2. Migrated DATR Cross-Domain Identification Performance

This subsection describes the experiments that show the performance of migrated DATR in the new environment.

**Figure 8:**
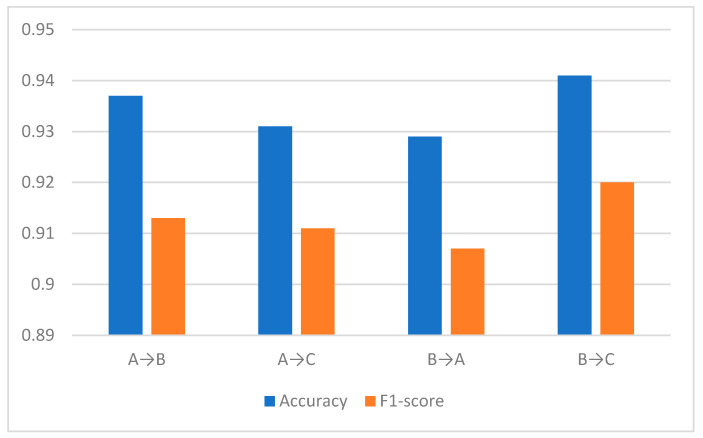
Experimental result.

The following four sets of transfer learning experiments are evaluated: dataset A → dataset B, dataset B → dataset A, dataset A → dataset C, and dataset B → dataset C. In front of the arrows are the source-domain datasets, and after the arrows are the target-domain datasets. The accuracy and F1 scores of the four transfer experiments are averaged over 10 experiments, as shown in Figure 8.

Comparing the experimental results, it can be seen that the DATR algorithm can improve the recognition accuracy of the recognition model on the target-domain vibration signal after transfer. Taking dataset A → dataset B as an example, the algorithm using transfer learning improves the recognition accuracy by 29.7% and the F1-score by 37% than that of the direct source-domain model, which indicates that the DATR algorithm has a better effect in moving ground-target recognition domain transfer. In addition, it can also be seen from the figure that all the recognition models obtained by DATR after transfer learning between different datasets have higher recognition accuracy than the model without transfer learning. This indicates that the algorithm has better constraints on extracting domain-invariant features, is able to extract common features of signals from different data domains in semi-supervised training with unlabeled data in the target domain, and has good target recognition performance.

#### 3.3.3. Visual Distribution of Different Algorithm Features

This subsection describes the experiments that show the feature distribution of different algorithms.

In order to observe the performance of the model after transfer learning more intuitively, the output features of the recognition algorithm are compared. The model output features are reduced from high-dimensional to two-dimensional using t-stochastic neighbor embedding (t-SNE). The visualization distributions of the features obtained from different recognition methods for processing vibration signals are compared.

**Figure 9:**
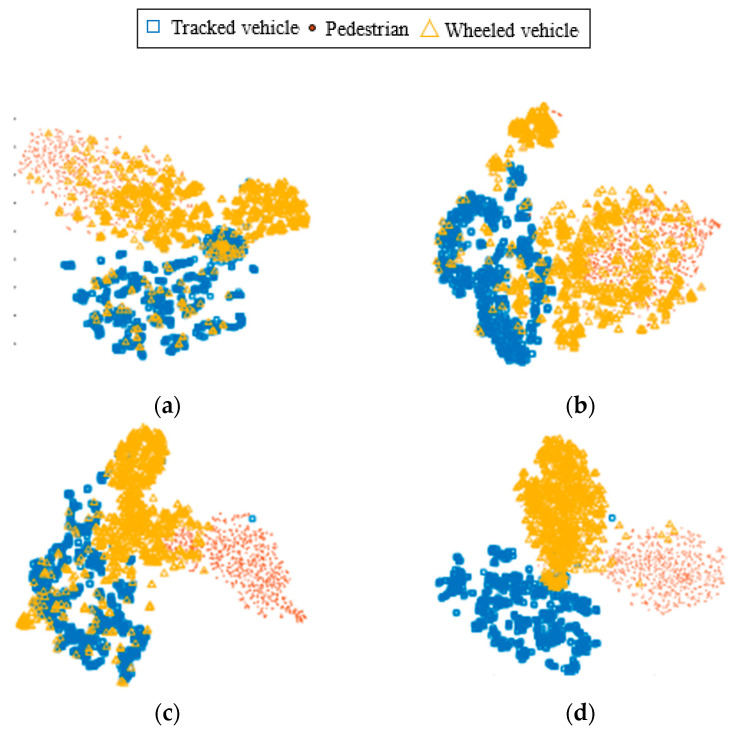
Feature visualization comparison: (**a**) VibCNN; (**b**) PRNN; (**c**) Unmigrated DATR; (**d**) Migrated DATR.

Specifically, model transfer from dataset A → B is performed using dataset A as the source-domain dataset and dataset B as the target-domain dataset. Compare the distribution of the output features of the feature extractor of the DATR algorithm after transfer with the distribution of the features of the unmigrated DATR algorithm (trained with dataset A only). Also, compare the distribution of features extracted by the VibCNN algorithm and the PRNN algorithm (trained with dataset A only) for dataset B recognition. The feature visualization results for each method are shown in Figure 9.

The red dots shown in Figure 9 are the visualized features of pedestrian vibration signals, the yellow triangles indicate the visualized features of wheeled vehicle vibration signals, and the blue squares indicate the visualized features of tracked vehicle vibration signals. Figure 9a–c show the feature visualization results of the VibCNN algorithm, PRNN algorithm and the DATR algorithm without using transfer learning. As can be seen from the figures, when the recognition algorithm without transfer learning trained on the source-domain data is used to discriminate the target-domain data, the intra-class feature distribution of the signals is relatively scattered and there is a large overlapping portion of inter-class features. This indicates that some of the recognizable features of the target-domain signals have changed, and the recognition model trained on the source-domain dataset cannot accurately determine the class of the target-domain signals.

Figure 9d shows the visualized clustering diagram of the features extracted from the target-domain data by the DATR algorithm after transfer learning. From the figure, it can be seen that although there is a small amount of feature overlap region in the center part of the visualized feature map, the overall distribution of features shows that the features extracted by the feature extractor have a good degree of intra-class aggregation and inter-class dispersion. This indicates that the signal model features after transfer learning can be able to extract features with separability from non-similar targets, and the unlabeled target-domain data can be used to train the target-domain recognition model and achieve target classification with higher accuracy.

#### 3.3.4. Visual Features of Different Domains

This subsection describes the experiments that show the ability of the proposed method to extract the common features of the source and target-domain data.

**Figure 10:**
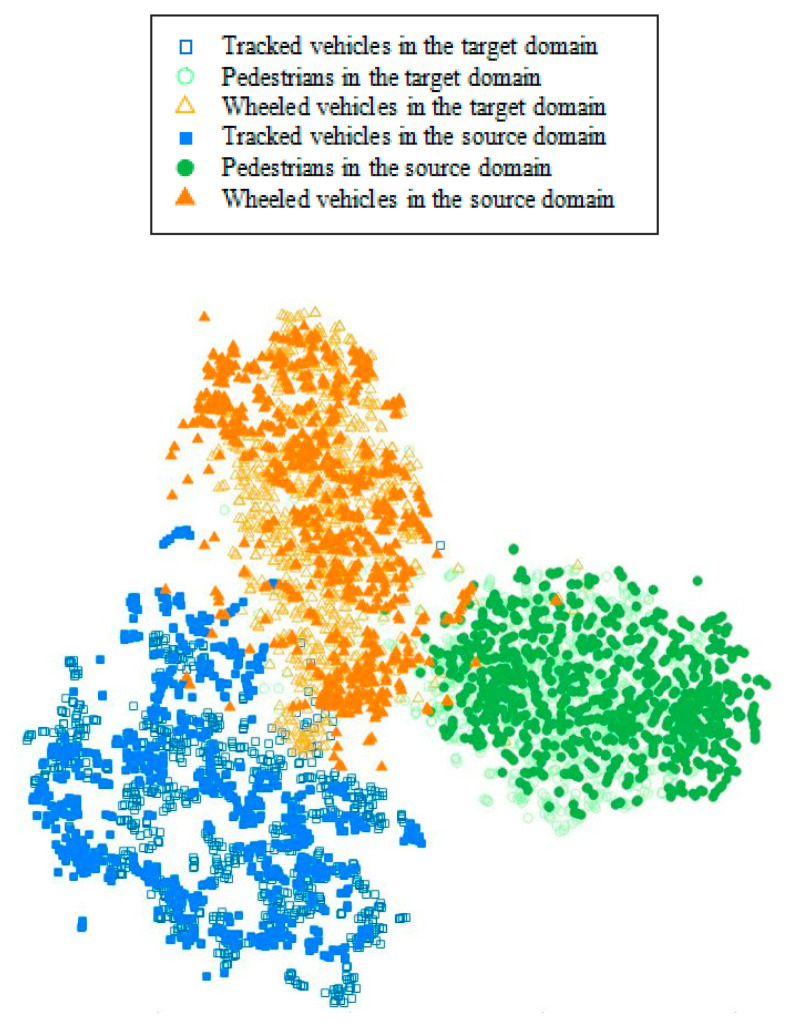
Feature visualization of the model after transfer learning.

The feature visualization results are shown in Figure 10. The hollow icons in the figure indicate the visualized features of the target domain and the solid icons indicate the visualized features of the source domain. From the figure, it can be seen that the distribution of features extracted by the model feature extractor from the same class of signals in the source and target domains is similar. As a whole, the features of the same class of targets in different domains are clustered in the same spatial range. This indicates that the DATR algorithm can extract domain-invariant features of the target vibration signals in the source domain and the target vibration signals in the target domain after transfer learning, and the proposed loss function can better constrain the training of the recognition model.

In summary, the neural network and domain adaptive moving ground-target recognition algorithm proposed in this paper can semi-supervise the training of target recognition models using source domain data and unlabeled target-domain data, and the trained models have better target recognition accuracy in both source and target domains.

## 4. Discussion, Limitation and Future Work

### 4.1. Discussion

In this paper, a moving ground-target recognition algorithm based on neural network and domain adaptive is proposed. The algorithm can improve the accuracy of recognition as well as solve the problem of degradation of the recognition model performance in new environment. The algorithm uses CNN to construct a feature extractor. Signal features can be extracted from a time-domain waveform and short-time Fourier transform, respectively. Input the feature into the target classifier and output the target classification result. At the same time, the algorithm uses transfer learning. The semi-supervised training of a target-domain recognition model can be realized. The algorithm can adapt to the change of environment. A joint loss function consisting of cross-entropy loss function, focal loss and MMD distance is proposed. The training of the target-domain feature extractor and target classifier can be constrained. The ability of feature extractor to extract domain invariant features in the time-domain and time–frequency domain is improved.

The target recognition algorithm proposed in this paper is compared with the comparison algorithm using different datasets. Experimental results show that the proposed target recognition algorithm can effectively shorten the training time and running time. The accuracy of moving ground-target type recognition is improved. At the same time, this domain-based adaptive algorithm does not need to label the vibration signal of the target domain. It can reduce the labor cost and time cost caused by data labeling, and has better adaptability.

### 4.2. Limitation and Future Work

The algorithm of moving ground-target recognition proposed in this paper is based on a vibration sensor. This reduces the power of the system and has the advantages of easy deployment, small size, and strong concealment. However, in harsh or new environments, vibration sensors-based recognition systems often show low detection accuracy and poor robustness. The algorithm proposed in this paper can significantly improve the recognition accuracy of new environment. However, in strong winds and heavy rain, the environmental noise increases. Vibration sensors may not detect moving ground targets.

Using multi-mode fusion technology to process the data obtained by various security equipment can make up for the defects of single-mode method. This method can improve the intelligence level of the field security system. However, there are some problems such as high power consumption, misalignment between modes, and complex model structure. This makes the field of multi-mode security detection has great limitations. Therefore, multi-modal data will be used in the future to improve the recognition accuracy of the system. How to fuse the feature information in multi-modal data is also studied. The algorithm can satisfy the security detection and identification tasks with low power consumption.

## Figures and Tables

**Table 1:** Number of samples in the dataset.

Dataset	Target Type	Number of Samples
Dataset A	Tracked vehicle signal in Liaoning Province	3000
	Wheeled vehicle signal in Liaoning Province	3000
	Pedestrian signal in Jilin Province	3000
	Environmental noise in Liaoning Province	3000
Dataset B	Tracked vehicle signal in Inner Mongolia	3000
	Wheeled vehicle signal in Chongqing	3000
	Pedestrian signal in Inner Mongolia	3000
	Environmental noise in Inner Mongolia	3000
Dataset C	Wheeled vehicle signal in Jiangsu Province	1000
	Pedestrian signal in Shanxi Province	1000
	Environmental noise in Shanxi Province	1000

**Table 2:** Model training parameter settings.

Parameter	Value
Learning rate	Initial 0.001
epoch	30
Batch size	128
Optimization function	Adam

**Table 3:** The performance of the three methods.

		DATR	VibCNN	PRNN
Accuracy	Validation set	0.964 ± 0.005	0.953 ± 0.016	0.937 ± 0.006
	Test set	0.962 ± 0.005	0.95 ± 0.017	0.934 ± 0.006
F1-score	Validation set	0.947 ± 0.007	0.936 ± 0.017	0.919 ± 0.008
	Test set	0.943 ± 0.006	0.932 ± 0.018	0.914 ± 0.006

**Table 4:** Comparison of training time and running time.

Classification Algorithm	DATR	VibCNN	PRNN
Training time	11 min 34 s	10 h 33 min 20 s	1 h 21 min 40 s
Run time	0.8 ms	4.6 ms	4 ms

## Data Availability

The data is unavailable due to privacy.
